# *catena*-Poly[[bis­(ethyl­enediamine)copper(II)]-μ-sulfato]

**DOI:** 10.1107/S160053681001737X

**Published:** 2010-05-19

**Authors:** Martin Lutz, Stef Smeets, Pascal Parois

**Affiliations:** a Bijvoet Center for Biomolecular Research, Crystal and Structural Chemistry, Faculty of Science, Utrecht University, Padualaan 8, 3584 CH Utrecht, The Netherlands

## Abstract

In the title compound, [Cu(SO_4_)(C_2_H_8_N_2_)_2_]_*n*_, the Cu, S and two O atoms lie on a mirror plane. The Cu atom is in a distorted octa­hedral environment and the ethyl­enediamine ligand is in a *gauche* conformation. The sulfate dianion is bridging, forming a one-dimensional chain. A two-dimensional net parallel to (001) is generated by N—H⋯O hydrogen bonding between the chains.

## Related literature

For related Cu(II) ethyl­enediamine complexes, see: Cullen & Lingafelter (1970 ▶); Bertini *et al.* (1979 ▶); Healy *et al.* (1978 ▶); Manriquez *et al.* (1996 ▶); Taylor *et al.* (2006 ▶). A similar variation of axial Cu—O distances is found in many weakly coord­inating anions such as sulfate (Castro *et al.*, 2002 ▶), nitrate (Plater *et al.*, 2008 ▶), perchlorate (Bernhardt *et al.*, 2001 ▶) or triflate (Liu *et al.*, 2007 ▶). The anisotropic mosaicity was treated according to Duisenberg (1983 ▶).
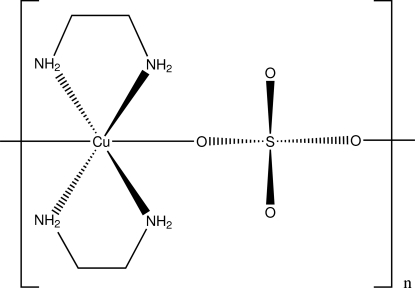


## Experimental

### 

#### Crystal data

[Cu(SO_4_)(C_2_H_8_N_2_)_2_]
*M* *_r_* = 279.81
Orthorhombic, 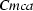

*a* = 14.4959 (3) Å
*b* = 9.63748 (8) Å
*c* = 13.87746 (17) Å
*V* = 1938.73 (5) Å^3^
*Z* = 8
Mo *K*α radiation
μ = 2.47 mm^−1^
*T* = 110 K
0.36 × 0.21 × 0.06 mm

#### Data collection

Nonius KappaCCD diffractometer
Absorption correction: analytical (*SADABS*; Sheldrick, 2008*a* ▶) *T*_min_ = 0.489, *T*_max_ = 0.910
29167 measured reflections
2204 independent reflections
1990 reflections with *I* > 2σ(*I*)
*R*_int_ = 0.032

#### Refinement

*R*[*F* ^2^ > 2σ(*F* ^2^)] = 0.019
*wR*(*F* ^2^) = 0.048
*S* = 1.10
2204 reflections
86 parameters
H atoms treated by a mixture of independent and constrained refinement
Δρ_max_ = 0.44 e Å^−3^
Δρ_min_ = −0.56 e Å^−3^

### 

Data collection: *COLLECT* (Nonius, 1999 ▶); cell refinement: *PEAKREF* (Schreurs, 2005 ▶); data reduction: *Eval15* (Schreurs *et al.*, 2010 ▶) and *SADABS* (Sheldrick, 2008*a* ▶); program(s) used to solve structure: *SHELXS97* (Sheldrick, 2008*b* ▶); program(s) used to refine structure: *SHELXL97* (Sheldrick, 2008*b* ▶); molecular graphics: *PLATON* (Spek, 2009 ▶); software used to prepare material for publication: *SHELXL97*.

## Supplementary Material

Crystal structure: contains datablocks I, global. DOI: 10.1107/S160053681001737X/vm2026sup1.cif

Structure factors: contains datablocks I. DOI: 10.1107/S160053681001737X/vm2026Isup2.hkl

Additional supplementary materials:  crystallographic information; 3D view; checkCIF report

## Figures and Tables

**Table:** 

Cu1—N1	2.0173 (8)
Cu1—N2	2.0226 (8)
Cu1—O1	2.3575 (9)
Cu1—O3^i^	2.4673 (9)

**Table:** 

N1^ii^—Cu1—N1	91.62 (4)
N1—Cu1—N2^ii^	176.81 (3)
N1—Cu1—N2	85.22 (3)
N2^ii^—Cu1—N2	97.95 (4)
N1—Cu1—O1	92.50 (3)
N2—Cu1—O1	87.27 (3)
N1—Cu1—O3^i^	92.95 (3)
N2—Cu1—O3^i^	87.59 (3)
O1—Cu1—O3^i^	172.18 (3)

**Table:** 

N1—C1—C2—N2	53.74 (10)

Symmetry codes: (i) 
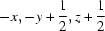
; (ii) 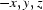.

**Table 2:** Hydrogen-bond geometry (Å, °)

*D*—H⋯*A*	*D*—H	H⋯*A*	*D*⋯*A*	*D*—H⋯*A*
N1—H1*N*⋯O2	0.858 (16)	2.280 (16)	3.0944 (11)	158.5 (14)
N1—H2*N*⋯O3^iii^	0.839 (17)	2.274 (17)	3.0642 (11)	157.1 (17)
N2—H3*N*⋯O2^iv^	0.845 (17)	2.125 (17)	2.9636 (10)	171.6 (16)
N2—H4*N*⋯O2^v^	0.859 (15)	2.210 (15)	3.0308 (10)	159.7 (14)

Symmetry codes: (iii) 
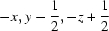
; (iv) 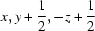; (v) 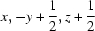.

**Fig. 1.**
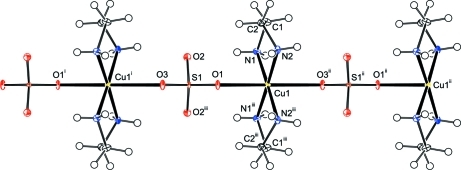
: Displacement ellipsoid plot of (I), drawn at the 50% probability level. Only three units of the polymeric chain are shown. View along the b axis. Symmetry operations i: -x, 0.5 - y, z - 1/2; ii: -x, 0.5 - y, z + 1/2; iii: -x, y, z.

**Fig. 2.**
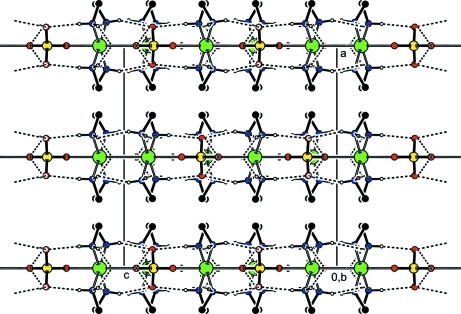
: Packing of (I) in the crystal viewed along the b axis. C—H hydrogen atoms are omitted for clarity. Hydrogen bonds are drawn as dashed lines.

**Fig. 3.**
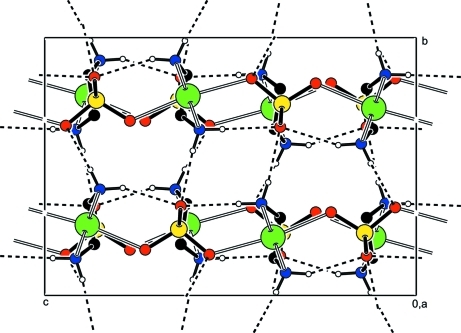
: Packing of (I) in the crystal viewed along the a axis. C—H hydrogen atoms are omitted for clarity. Hydrogen bonds are drawn as dashed lines.

## supplementary crystallographic information

### Comment

Ethylenediamine (en) complexes of transition metals belong to the most studied compounds in inorganic chemistry. In the case of copper sulfate the tris(ethylenediamine) complex is known at room temperature (Cullen & Lingafelter, 1970) as well as at 120 K after a solid-solid phase transition (Bertini *et al.*, 1979). These crystal structures show the copper in an octahedral geometry and the sulfate is not coordinated to the metal but hydrogen bonded to the amine groups.

Here, we report the crystal structure of the bis(ethylenediamine) complex of copper sulfate (I), in which the sulfate is bridging two copper centers. The compound thus forms a polymeric chain by coordination, which runs in the direction of the *c* axis (Fig. 1). The copper, the sulfur and two O atoms are in special positions on the crystallographic mirror plane of the orthorhombic space group Cmca. Bridging sulfate ions are very common in copper complexes. For example, in [Cu(en)(H_2_O)_2_]SO_4_, the sulfate is a bridging ligand and a one-dimensional chain is formed by coordination (Healy *et al.*, 1978; Manriquez *et al.*, 1996; Taylor *et al.*, 2006).

As expected, the ethylenediamine ligand is in a *gauche* conformation (Table 1) and the copper is in a distorted octahedral environment. The nitrogen atoms form the equatorial plane with Cu—N distances (2.0173 (8) and 2.0226 (8) Å), which are shorter than in the room temperature structure of the tris(ethylenediamine) complex (2.150 (2) Å). The two axial positions are occupied by oxygen atoms of sulfate anions with much longer distances than in the equatorial plane. This can be explained by the Jahn-Teller effect in Cu(II) compounds. The Cu—O distances (2.3575 (9) and 2.4673 (9) Å) also differ significantly compared to each other. Such differences are not uncommon with copper complexes of weakly coordinating anions like sulfate (Castro *et al.*, 2002), nitrate (Plater *et al.*, 2008), perchlorate (Bernhardt *et al.*, 2001) or trifluoromethanesulfonate (Liu *et al.*, 2007).

The coordinated NH_2_ groups act as hydrogen bond donors and the non-coordinated sulfate O-atoms act as acceptors (Table 2). Two hydrogen bonds are formed within the coordination polymer *via* H1N and H4N. Two hydrogen bonds *via* H2N and H3N are between the chains resulting in a two-dimensional net in the b,c-plane (Figures 2 and 3). This two-dimensional motif is also reflected in the plate shaped crystal habitus, where the a-direction has the smallest dimension.

### Experimental

2.04 g of copper sulfate pentahydrate (8.17 mmol) were dissolved in 150 ml of water and brought to boiling temperature. Then 2 ml of ethylenediamine (37 mmol) were added dropwise. The resulting deep blue solution was concentrated at 333 K and atmospheric pressure. In the concentrated solution, crystals appeared after 2 days of evaporation at room temperature.

### Refinement

An anisotropic mosaic model was used in the intensity integration with *hkl* = (0,0,1) as anisotropic vector (Duisenberg, 1983). Hydrogen atoms were located in difference Fourier maps. N—H hydrogen atoms were refined freely with isotropic displacement parameters. C—H hydrogen atoms were refined using a riding model with C—H = 0.99 Å and with *U*_iso_(H) = 1.2 times *U*_eq_(C).

### Crystal data

**Table:**

[Cu(SO_4_)(C_2_H_8_N_2_)_2_]	*F*(000) = 1160
*M**_r_* = 279.81	*D*_x_ = 1.917 Mg m^−^^3^
Orthorhombic, *C**m**c**a*	Mo *K*α radiation, λ = 0.71073 Å
Hall symbol: -C 2bc 2	Cell parameters from 21936 reflections
*a* = 14.4959 (3) Å	θ = 2.5–35.0°
*b* = 9.63748 (8) Å	µ = 2.47 mm^−^^1^
*c* = 13.87746 (17) Å	*T* = 110 K
*V* = 1938.73 (5) Å^3^	Plate, blue
*Z* = 8	0.36 × 0.21 × 0.06 mm

### Data collection

**Table:**

Nonius KappaCCD diffractometer	2204 independent reflections
Radiation source: rotating anode	1990 reflections with *I* > 2σ(*I*)
graphite	*R*_int_ = 0.032
φ and ω scans	θ_max_ = 35.0°, θ_min_ = 2.8°
Absorption correction: analytical (*SADABS*; Sheldrick, 2008a)	*h* = −23→23
*T*_min_ = 0.489, *T*_max_ = 0.910	*k* = −15→15
29167 measured reflections	*l* = −20→22

### Refinement

**Table:**

Refinement on *F*^2^	Primary atom site location: structure-invariant direct methods
Least-squares matrix: full	Secondary atom site location: difference Fourier map
*R*[*F*^2^ > 2σ(*F*^2^)] = 0.019	Hydrogen site location: difference Fourier map
*wR*(*F*^2^) = 0.048	H atoms treated by a mixture of independent and constrained refinement
*S* = 1.10	*w* = 1/[σ^2^(*F*_o_^2^) + (0.0208*P*)^2^ + 2.1845*P*] where *P* = (*F*_o_^2^ + 2*F*_c_^2^)/3
2204 reflections	(Δ/σ)_max_ = 0.001
86 parameters	Δρ_max_ = 0.44 e Å^−^^3^
0 restraints	Δρ_min_ = −0.56 e Å^−^^3^

### Special details

**Table:**

Geometry. All esds (except the esd in the dihedral angle between two l.s. planes) are estimated using the full covariance matrix. The cell esds are taken into account individually in the estimation of esds in distances, angles and torsion angles; correlations between esds in cell parameters are only used when they are defined by crystal symmetry. An approximate (isotropic) treatment of cell esds is used for estimating esds involving l.s. planes.
Refinement. Refinement of *F*^2^ against ALL reflections. The weighted *R*-factor wR and goodness of fit *S* are based on *F*^2^, conventional *R*-factors *R* are based on *F*, with *F* set to zero for negative *F*^2^. The threshold expression of *F*^2^ > σ(*F*^2^) is used only for calculating *R*-factors(gt) etc. and is not relevant to the choice of reflections for refinement. *R*-factors based on *F*^2^ are statistically about twice as large as those based on *F*, and *R*- factors based on ALL data will be even larger.

### Fractional atomic coordinates and isotropic or equivalent isotropic displacement parameters (Å^2^)

**Table:**

	*x*	*y*	*z*	*U*_iso_*/*U*_eq_	
Cu1	0.0000	0.226359 (15)	0.385553 (11)	0.00587 (4)	
S1	0.0000	0.24252 (3)	0.13888 (2)	0.00541 (5)	
O1	0.0000	0.32265 (9)	0.22939 (6)	0.00932 (16)	
O2	0.08343 (5)	0.15354 (7)	0.13383 (5)	0.01049 (12)	
O3	0.0000	0.34145 (9)	0.05699 (7)	0.00990 (16)	
N1	0.09978 (5)	0.08890 (8)	0.35157 (6)	0.00890 (13)	
H1N	0.0983 (10)	0.0830 (15)	0.2899 (12)	0.018 (4)*	
H2N	0.0882 (13)	0.0121 (18)	0.3774 (10)	0.022 (4)*	
N2	0.10526 (6)	0.35659 (8)	0.41674 (6)	0.00842 (12)	
H3N	0.0944 (12)	0.4387 (18)	0.3986 (11)	0.020 (4)*	
H4N	0.1142 (11)	0.3552 (15)	0.4779 (11)	0.016 (4)*	
C1	0.18950 (6)	0.14531 (9)	0.38195 (7)	0.01121 (15)	
H1A	0.2397	0.1034	0.3434	0.013*	
H1B	0.2007	0.1241	0.4508	0.013*	
C2	0.18704 (7)	0.30147 (9)	0.36647 (7)	0.01045 (15)	
H2A	0.2438	0.3445	0.3926	0.013*	
H2B	0.1833	0.3227	0.2968	0.013*	

### Atomic displacement parameters (Å^2^)

**Table:**

	*U*^11^	*U*^22^	*U*^33^	*U*^12^	*U*^13^	*U*^23^
Cu1	0.00700 (7)	0.00409 (6)	0.00652 (7)	0.000	0.000	−0.00089 (4)
S1	0.00864 (12)	0.00388 (10)	0.00371 (11)	0.000	0.000	−0.00008 (8)
O1	0.0172 (4)	0.0071 (4)	0.0036 (4)	0.000	0.000	−0.0017 (3)
O2	0.0111 (3)	0.0101 (3)	0.0102 (3)	0.0040 (2)	0.0003 (2)	−0.0006 (2)
O3	0.0192 (4)	0.0057 (3)	0.0048 (4)	0.000	0.000	0.0012 (3)
N1	0.0094 (3)	0.0064 (3)	0.0109 (3)	−0.0003 (2)	−0.0013 (3)	−0.0010 (2)
N2	0.0126 (3)	0.0064 (3)	0.0063 (3)	−0.0012 (2)	0.0005 (2)	−0.0004 (2)
C1	0.0091 (4)	0.0088 (3)	0.0157 (4)	0.0001 (3)	−0.0027 (3)	−0.0002 (3)
C2	0.0103 (4)	0.0094 (3)	0.0116 (4)	−0.0024 (3)	0.0012 (3)	−0.0003 (3)

### Geometric parameters (Å, °)

**Table:**

Cu1—N1^i^	2.0172 (8)	N1—H1N	0.858 (16)
Cu1—N1	2.0173 (8)	N1—H2N	0.839 (17)
Cu1—N2^i^	2.0226 (8)	N2—C2	1.4745 (12)
Cu1—N2	2.0226 (8)	N2—H3N	0.845 (17)
Cu1—O1	2.3575 (9)	N2—H4N	0.859 (15)
Cu1—O3^ii^	2.4673 (9)	C1—C2	1.5207 (13)
S1—O1	1.4745 (9)	C1—H1A	0.9900
S1—O3	1.4834 (9)	C1—H1B	0.9900
S1—O2^i^	1.4842 (7)	C2—H2A	0.9900
S1—O2	1.4842 (7)	C2—H2B	0.9900
N1—C1	1.4712 (12)		
			
N1^i^—Cu1—N1	91.62 (4)	C1—N1—H1N	109.4 (10)
N1^i^—Cu1—N2^i^	85.22 (3)	Cu1—N1—H1N	105.0 (10)
N1—Cu1—N2^i^	176.81 (3)	C1—N1—H2N	112.3 (12)
N1^i^—Cu1—N2	176.81 (3)	Cu1—N1—H2N	109.6 (12)
N1—Cu1—N2	85.22 (3)	H1N—N1—H2N	111.3 (14)
N2^i^—Cu1—N2	97.95 (4)	C2—N2—Cu1	106.36 (5)
N1^i^—Cu1—O1	92.50 (3)	C2—N2—H3N	110.2 (11)
N1—Cu1—O1	92.50 (3)	Cu1—N2—H3N	112.1 (11)
N2^i^—Cu1—O1	87.27 (3)	C2—N2—H4N	110.0 (10)
N2—Cu1—O1	87.27 (3)	Cu1—N2—H4N	108.4 (10)
N1^i^—Cu1—O3^ii^	92.95 (3)	H3N—N2—H4N	109.7 (14)
N1—Cu1—O3^ii^	92.95 (3)	N1—C1—C2	107.73 (7)
N2^i^—Cu1—O3^ii^	87.59 (3)	N1—C1—H1A	110.2
N2—Cu1—O3^ii^	87.59 (3)	C2—C1—H1A	110.2
O1—Cu1—O3^ii^	172.18 (3)	N1—C1—H1B	110.2
O1—S1—O3	108.42 (5)	C2—C1—H1B	110.2
O1—S1—O2^i^	110.05 (3)	H1A—C1—H1B	108.5
O3—S1—O2^i^	109.58 (3)	N2—C2—C1	107.97 (7)
O1—S1—O2	110.05 (3)	N2—C2—H2A	110.1
O3—S1—O2	109.59 (3)	C1—C2—H2A	110.1
O2^i^—S1—O2	109.14 (6)	N2—C2—H2B	110.1
S1—O1—Cu1	125.23 (5)	C1—C2—H2B	110.1
C1—N1—Cu1	108.92 (5)	H2A—C2—H2B	108.4
			
O3—S1—O1—Cu1	180.0	N2—Cu1—N1—C1	9.25 (6)
O2^i^—S1—O1—Cu1	60.16 (3)	O1—Cu1—N1—C1	96.30 (6)
O2—S1—O1—Cu1	−60.16 (3)	N1—Cu1—N2—C2	19.71 (6)
N1^i^—Cu1—O1—S1	−45.86 (2)	N2^i^—Cu1—N2—C2	−159.89 (4)
N1—Cu1—O1—S1	45.86 (2)	O1—Cu1—N2—C2	−73.03 (6)
N2^i^—Cu1—O1—S1	−130.95 (2)	Cu1—N1—C1—C2	−35.63 (9)
N2—Cu1—O1—S1	130.95 (2)	Cu1—N2—C2—C1	−44.31 (8)
N1^i^—Cu1—N1—C1	−171.12 (4)	N1—C1—C2—N2	53.74 (10)

Symmetry codes: (i) −*x*, *y*, *z*; (ii) −*x*, −*y*+1/2, *z*+1/2.

### Hydrogen-bond geometry (Å, °)

**Table:**

*D*—H···*A*	*D*—H	H···*A*	*D*···*A*	*D*—H···*A*
N1—H1N···O2	0.858 (16)	2.280 (16)	3.0944 (11)	158.5 (14)
N1—H2N···O3^iii^	0.839 (17)	2.274 (17)	3.0642 (11)	157.1 (17)
N2—H3N···O2^iv^	0.845 (17)	2.125 (17)	2.9636 (10)	171.6 (16)
N2—H4N···O2^v^	0.859 (15)	2.210 (15)	3.0308 (10)	159.7 (14)

Symmetry codes: (iii) −*x*, *y*−1/2, −*z*+1/2; (iv) *x*, *y*+1/2, −*z*+1/2; (v) *x*, −*y*+1/2, *z*+1/2.
